# Exploring autism spectrum profiles via network analysis of parent-reported ASSQ patterns

**DOI:** 10.1186/s12888-026-07935-z

**Published:** 2026-03-05

**Authors:** Mingwan Zhou, Honghe Zhang

**Affiliations:** Psychological Assessment Center, Xiamen Xianyue Hospital, Xianyue Hospital Affiliated with Xiamen Medical College, Fujian Psychiatric Center, Fujian Clinical Research Center for Mental Disorders, No. 387, Xianyue Road, Siming District, Xiamen, Fujian Province 360102 China

**Keywords:** Autism spectrum disorder, ASSQ, Network analysis, Children and adolescents

## Abstract

**Background:**

The behavioral profile of autism spectrum disorder (ASD) is highly heterogeneous. Analyses relying on total scores often fail to capture the associative patterns among individual behavioral items. This study applied network analysis to parent-reported Autism Spectrum Screening Questionnaire (ASSQ) data to explore the internal associative structure among core ASD behavioral domains and to identify key items within the network.

**Methods:**

This retrospective study included 995 children and adolescents aged 7–16 years diagnosed with ASD according to ICD-10 criteria at Xiamen Xianyue Hospital between 2019 and 2025, whose parents completed the ASSQ. Given the 3-point Likert scale of the ASSQ, two parallel network modeling strategies were employed to enhance robustness: a dichotomized (Ising model) and an ordinal (Gaussian Graphical Model, GGM) approach to explore associations among ASSQ items. Comorbidities and psychiatric medication history were included as covariates. Central and bridge nodes were identified using expected influence and bridge expected influence, respectively, and the optimal model was selected. Network Comparison Tests were conducted to examine differences in global strength and structure between sex (male/female) and age groups (7–11/12–16 years).

**Results:**

(1) The ordinal network model (ASSQ-GGM network) demonstrated better centrality stability and was therefore selected for reporting. (2) The ASSQ-GGM network identified the following central nodes: Q11 (inability to adjust, EI = 1.238), Q27 (unusual posture, EI = 1.154), and Q15 (fails to make relationships with peers, EI =  1.047). Bridge nodes were: Q26 (unusual facial expression, bEI = 0.806), Q11 (inability to adjust, bEI = 0.719) and Q12 (lacks empathy, bEI=0.710). (3) Network Comparison Tests found no significant differences in network structure by sex or age, which may be due to insufficient or imbalanced subsample sizes. The possibility of sex- or age-specific network patterns cannot be ruled out.

**Conclusions:**

This study presents a network of interrelated parent-reported ASD-related behavioral features based on ASSQ items. No significant sex or age differences in network structure were found. These findings provide insights into the associative patterns among ASSQ items. As this study is based on cross-sectional, guardian-reported data, the findings are hypothesis-generating and do not support causal inferences. Any implications for intervention require validation through longitudinal or experimental studies.

**Clinical trial number:**

Not applicable.

**Supplementary Information:**

The online version contains supplementary material available at 10.1186/s12888-026-07935-z.

## Background

Autism spectrum disorder (ASD) is a neurodevelopmental condition characterized by deficits in social communication and interaction, alongside restricted and repetitive patterns of behavior [1]. Although diagnostic criteria are well-established, ASD individuals exhibit substantial heterogeneity in symptom manifestation, severity, and comorbidities, posing fundamental challenges to understanding its underlying mechanisms and enabling precise assessment and intervention [2–4]. Traditional research has largely relied on comparisons of total scores or factor scores between groups. While this approach can identify group differences, it fails to reveal the complex network of interactions among the specific symptoms constituting the clinical presentation of ASD. In recent years, the psychopathological network theory, originating from complex systems science, has provided a novel perspective for understanding mental disorders. This theory posits that a disorder is not caused by a single latent variable but is rather represented and maintained by a network of direct, mutually reinforcing causal associations among symptoms [5, 6]. Within this framework, identifying core symptoms with high influence within the network, and bridge symptoms that connect different symptom clusters, is crucial for understanding the disorder’s pathological structure and potential intervention targets. Network analytic methods have been successfully applied across various mental disorders [7, 8].

However, existing network analysis studies focusing on ASD have primarily centered on two areas: investigating the associative networks between core ASD traits and comorbid symptoms (e.g., anxiety [9, 10], depression [11], irritability [12]), and conducting network analyses based on a limited number of items from diagnostic observation tools (e.g., ADOS [13]) or specific social-cognitive tasks [14]. While valuable, these studies have generally not systematically explored the interactive patterns *within* and *between* the three core ASD behavioral domains as defined by ICD-10—social interaction deficits, communication deficits, and restricted, repetitive behaviors—at the level of specific behavioral traits. Therefore, whether these core behavioral domains are relatively independent in phenomenology or possess intrinsic connecting pathways remains an important empirical question.

This study employs the Chinese version of the Autism Spectrum Screening Questionnaire (ASSQ) [15, 16] as the analytical vehicle. The ASSQ is a well-established screening instrument for autism spectrum-related behavioral traits and is one of the most widely used tools for identifying children and adolescents aged 7–16 who may have ASD [15]. Its 27 items are rated on a 3-point scale, and its dimensions [16] (social interaction, communication problems, and restricted repetitive behaviors) broadly correspond to the three core diagnostic domains for autism in ICD-10. The ASSQ was selected primarily for its strong psychometric properties [17]: it has demonstrated a high positive predictive value for ASD diagnosis (up to 90%), excellent test-retest reliability (*r* = 0.90, *p* < 0.001), good inter-rater reliability between parents and teachers (*r* = 0.90, *p* < 0.001), and high sensitivity (91%) and specificity (86%). Furthermore, the ASSQ has demonstrated good cross-cultural applicability, having been validated in diverse cultural contexts including Japan [18], Finland [19], Turkey [20], and Georgia [21].

Based on this, the aims of this study in a sample of children and adolescents with ASD are: (1) To construct and describe the network of ASD behavioral traits based on parent-reported ASSQ items and characterize its overall structure. (2) To identify core traits: determining the core behavioral traits that exert the greatest centrality influence within the network and may be pivotal for network stability. (3) To examine dimensional associations and explore bridging pathways: although ASSQ items are categorized into theoretical dimensions (social, communication, restricted repetitive behaviors), these dimensions may not be independent at the behavioral level. This study will employ network analysis to exploratorily identify key “bridge nodes” that potentially connect different dimensions. As an exploratory, cross-sectional analysis, the primary purpose of this study is to generate hypotheses regarding the network structure and the mechanisms of connectivity between domains based on the co-occurrence patterns of parent-reported ASD-related behavioral traits on the ASSQ, providing a new perspective and preliminary basis for future, more in-depth research.

## Methods

### Participants

This retrospective study included patients with Autism Spectrum Disorder(ASD) who visited Xiamen Xianyue Hospital between 2019 and 2025. Data were extracted from comprehensive clinical electronic medical records. Participants had to meet the following inclusion criteria: (1) children and adolescents aged 7–16 years; (2) diagnosed with ASD by a psychiatrist based on ICD-10 criteria (including childhood autism, atypical autism, and Asperger’s syndrome) as documented in the clinical electronic medical record system; (3) completion of the Autism Spectrum Screening Questionnaire (ASSQ) by the child’s parent (primary caregiver) during the initial assessment at the hospital’s psychological assessment center, with assistance from a professionally trained psychological assessor. For patients with multiple assessments, the ASSQ completed by the parent during the first visit was used. A total of 995 patients diagnosed with ASD were ultimately included in the study. The demographic and clinical characteristics of the included patients are presented in Table 1. The main variables were defined as follows: (1) Comorbid diagnoses: Patients with only an ASD diagnosis (childhood autism, atypical autism, or Asperger’s syndrome) were classified as having a single diagnosis. For other patients, comorbid conditions were categorized based on medical records into the following groups: mood disorders, attention deficit hyperactivity disorder (ADHD), obsessive-compulsive disorder, sleep disorders, tic disorders, oppositional defiant disorder/conduct disorder, somatic symptom disorders, and eating disorders. (2) History of psychiatric medication: Due to the complexity of actual medication records involving various drugs, dosages, and treatment regimens, medication use in this study was simplified to a binary variable (yes/no). In this sample, 704 patients (70.8%) had a history of psychiatric medication use. (3) Intellectual level: Assessed using the Wechsler Intelligence Scale for Children, Fourth Edition (WISC-IV), applicable to individuals aged 6–16 years. In clinical practice, this assessment is performed based on the physician’s clinical judgment regarding its necessity, and not all patients undergo it. In this study, 376 patients had complete intellectual test results (data missingness rate: 62.21%). Given the high missingness rate, intellectual level was reported only as a descriptive variable to characterize its distribution and was not included in subsequent model analyses. Comorbid diagnoses and history of psychiatric medication were included as covariates in the main network analysis. The study protocol was reviewed and approved by the Ethics Review Committee of Xiamen Xianyue Hospital (Approval No. 2024-KY-099). As a retrospective study, written informed consent from participants was waived in accordance with hospital ethics committee regulations.

**Table 1:** Basic demographic characteristics

Diagnosis	*N*	Gender (Male/Famale)	Age (M ± SD)	Psychiatric Medication (%)	IQ	
					Total/ Abnormal	(M ± SD)
Total	995	715/280	10.77 ± 3.43	704(70.8%)	376/72	82.88 ± 18.78
ASD Only	617	477/140	9.93 ± 3.49	376(60.9%)	224/47	80.69 ± 19.46
Comorbid Mood Disorder	170	76/94	13.36 ± 2.14	154(90.6%)	51/5	89.29 ± 14.45
Comorbid ADHD	131	114/17	9.94 ± 2.64	107(81.7%)	83/18	81.71 ± 19.83
Comorbid Obsessive-Compulsive Disorder	25	15/10	13.32 ± 1.86	23(92.0%)	7/1	94.17 ± 19.98
Comorbid Sleep Disorder	23	11/12	14.04 ± 1.30	21(91.3%)	7/1	93.86 ± 15.58
Comorbid Tic Disorder	11	10/1	12.36 ± 2.80	10(90.9%)	3/0	90.00 ± 3.00
Comorbid Oppositional Defiant Disorder / Conduct Disorder	9	8/1	12.00 ± 2.69	8(88.9%)	1/0	/
Comorbid Somatic Symptom Disorder	6	3/3	12.50 ± 2.81	3(50.0%)	/	/
Comorbid Eating Disorder	3	1/2	14.67 ± 2.31	2(66.7%)	/	/

Note: Based on ICD-10 diagnoses, patients diagnosed solely with childhood autism, atypical autism, or Asperger’s syndrome were defined as having a single diagnosis in this study. Due to the complexity of actual medication records involving various drugs, dosages, and treatment regimens, medication use was simplified to whether psychiatric medication was prescribed. Intelligence level: Only a subset of patients completed the Wechsler Intelligence Scale for Children. For each diagnostic category, the table presents the total number of patients who underwent intelligence testing and the number with abnormal intelligence (score below 70). “Comorbid Attention-Deficit/Hyperactivity Disorder” is abbreviated as comorbid ADHD

### Measurement

#### Autism spectrum screening questionnaire (ASSQ)

The ASSQ was originally developed by Ehlers et al. [15] and translated into Chinese by Guo Yanqing et al. [16]. This parent-report questionnaire is intended for children aged 7–16 years and can be used to screen school-aged children with Asperger’s syndrome or ASD who have normal or mild intellectual disability. It consists of 27 items divided into three dimensions [16]: social interaction (items 1, 12, 14, 15, 16, 17, 19, 25, 26), communication problems (items 4, 5, 6, 7, 8, 11, 13) and restricted and repetitive behaviors (items 2, 3, 9, 10, 18, 20, 21, 22, 23, 24, 27). Items are rated on a 3-point Likert scale (0 = normal, 1 = some problems, 2 = definite problems). A total score > 19 suggests the child may have autism spectrum disorder and should be referred to a professional for further detailed assessment and diagnosis. In the current study, the Cronbach’s alpha coefficient for this scale was 0.905.

### Data analysis

#### Descriptive statistics and network estimation

Descriptive statistical analyses were performed using SPSS software (version 28.0). All network analyses were conducted in the R environment (version 4.2.1).

### Covariate control and data preprocessing

To control for potential confounding effects of comorbid diagnoses and psychiatric medication history, tailored covariate adjustment methods were applied for the two network models. Both covariates were coded as binary variables: (1) comorbidity status: 0 = no comorbidity, 1 = presence of comorbidity (including mood disorders, ADHD, OCD, sleep disorders, tic disorders, oppositional defiant disorder/conduct disorder, somatic symptom disorder, or eating disorders); and (2) medication history: 0 = no, 1 = yes. For the Gaussian graphical model (GGM), a linear regression residualization approach was employed. For each of the 27 ASSQ items, a linear model was fitted with comorbidity and medication as predictors, and the standardized residuals were extracted. Correlation analysis between the residuals and covariates showed that all absolute correlation coefficients were below 0.001, indicating that the linear effects of the covariates were successfully removed. These residuals constituted the dataset for GGM estimation. For the Ising model, a logistic regression residualization approach was used. First, for each binarized ASSQ item, a logistic regression model was fitted with comorbidity and medication as predictors, and deviance residuals were extracted. These residuals were then re-binarized using a median split to obtain the final dataset for Ising network estimation. To evaluate the effectiveness of covariate adjustment, we compared network structures with and without covariate control. The correlation of edge weights was r = 0.734, indicating that covariates had a substantial impact on network structure and that controlling for them was necessary. All subsequent network analyses were conducted based on these residualized datasets. Therefore, the estimated network edges represent conditional associations among ASSQ items after removing linear and logistic effects of comorbidity and medication history.

### Network estimation

To explore the interactions among items of the Autism Spectrum Screening Questionnaire (ASSQ), networks were constructed using the preprocessed data described above, with the 27 items serving as nodes. Given the ASSQ’s 3-point Likert scale responses (0 = “normal”, 1 = “some problems”, 2 = “definite problems”), two parallel analytic strategies were employed to ensure robustness of the findings. The first strategy involved a dichotomized network (referred to as the ASSQ-Ising network): Following prior research [22], responses were recoded with “normal” as 0 (indicating “no symptom”) and “some problems” and “definite problems” combined as 1 (indicating “symptom present”). The network was fitted using the IsingFit package, employing the Extended Bayesian Information Criterion (EBIC) together with Least Absolute Shrinkage and Selection Operator (LASSO) regularization (gamma = 0.25) to automatically select the optimal sparse network, thereby effectively controlling for spurious associations [23, 24]. Given the sensitivity of the Ising model to the prevalence of binary data, items with high prevalence may exhibit spuriously high centrality (pseudo-core) due to low thresholds. To examine this potential bias, we first reported the positive proportion (proportion coded “1”) for each item and calculated the correlation between this proportion and the EI and bEI values from the Ising model. The second strategy involved an ordinal network analysis (referred to as the ASSQ-GGM network), which retained the original 3-level scores. A polychoric correlation matrix was computed using the psych package. A Gaussian Graphical Model (GGM) was then fitted using the EBICglasso() function from the qgraph package [25, 26], with the hyperparameter gamma set to 0.25. To assess the stability of the results, and in line with Epskamp et al. [25] who suggest a gamma range of [0, 0.5], we report in the Supplementary Materials the correlation stability (CS) coefficients for EI and bEI across both network structures with gamma values of 0, 0.25, and 0.5.

### Network visualization and metric calculation

Node placement in all network visualizations was determined using the Fruchterman-Reingold algorithm [27]: nodes with stronger or more connections are positioned closer together, meaning highly connected nodes tend to cluster near the center while others are distributed towards the periphery. Node centrality was measured using Expected Influence (EI). The node with the highest EI is considered the central node of the network [28], typically indicating its important position within the network’s interconnectivity pattern. Furthermore, to investigate interactions among the three ASSQ dimensions (social interaction, communication problems, and restricted repetitive behaviors), bridge centrality analysis was performed. Bridge centrality metrics, specifically bridge Expected Influence (bEI), were calculated using the bridge function from the Networktools package [29]. The node with the highest bEI is defined as the key bridge node, potentially serving as a hub connecting different symptom dimensions.

### Network centrality and stability assessment

To ensure the reliability of the above findings, we employed the bootnet package to assess the stability and accuracy of the network [30]. Node stability within the network structure of this study was evaluated using 1000 bootstrap samples via the case-dropping bootstrap, and the stability of EI and bEI was quantified by calculating the corresponding stability coefficients. A CS coefficient > 0.50 indicates good stability, while a value ≥ 0.25 is considered acceptable [25]. Edge accuracy was assessed by calculating 95% confidence intervals for edges via nonparametric bootstrap (1000 iterations); narrower intervals denote higher estimation precision [30].

### Model selection and validation

To determine the final network structure for reporting, the ASSQ-Ising network and the ASSQ-GGM network were systematically compared based on the following predefined metrics:1. Node Centrality Stability: The correlation stability (CS) coefficients for EI and bEI were calculated and compared for both networks. A CS coefficient > 0.5 indicates good stability, while a value ≥ 0.25 is considered acceptable [25]. 2. Edge Weight Accuracy: The width of the 95% confidence intervals (CIs) for edge weights, based on 1000 bootstrap samples, was examined. Additionally, two derived metrics were computed: (a) The proportion of edges with “acceptable” estimation precision, defined as edges whose CI width is ≤ |edge weight| × 0.5; (b) The proportion of statistically significant edges, defined as edges whose 95% CI does not include zero, relative to the total number of estimated edges. Model selection followed a predefined decision process: First, networks with CS coefficients < 0.25 were excluded. Among networks with CS coefficients ≥ 0.5, priority was given to the network demonstrating superior edge weight stability and accuracy. All comparative results are presented in full in the Supplementary Materials. The selected model was then used for all subsequent statistical analyses.

### Network comparison

To examine whether the ASSQ symptom network structure exhibits statistically significant gender or age differences, we employed the NetworkComparisonTest package to conduct a network comparison test (NCT). This test utilizes 1000 Bootstrap samples and a significance level of α = 0.05 to assess the following three levels [31]: Global Strength: Compares whether the total sum of absolute values of all edge weights differs between the two networks; Network Structure: Tests whether the distribution patterns of all edge weights across the two networks are identical; Specific Edge Strength: Examines whether the weight of each individual edge differs significantly between the two groups. Setting the maximum parameter to TRUE employs the maximum absolute value of the test statistic as the overall test statistic, thereby providing a more conservative global p-value that accounts for multiple comparisons.

## Results

### Descriptive statistics

Demographic and clinical characteristics of the patients are summarized in Table 1. For statistical analysis, comorbidity status was recoded as a binary variable: 0 = no comorbidity, 1 = presence of comorbidity (including mood disorders, ADHD, OCD, sleep disorders, tic disorders, ODD/CD, somatic symptom disorder, or eating disorders). Psychiatric medication history (0 = no, 1 = yes) was also included as a covariate in subsequent analyses. Intellectual level was not included in the main analysis due to a high rate of missing data (62.21%). For clarity in reporting, items of the Autism Spectrum Screening Questionnaire (ASSQ) are referred to as Q1 through Q27. Abbreviated labels for each item were established based on a review of relevant literature and consensus among the research team (see Supplementary Table S1 for details).

### Network analysis of the ASSQ network

#### Network estimation of the ASSQ network

Both analytic strategies constructed networks using the 27 ASSQ items as nodes. The potential confounding effects of comorbid diagnoses and psychiatric medication history were controlled for via a residualization preprocessing method. 

**Fig. 1:**
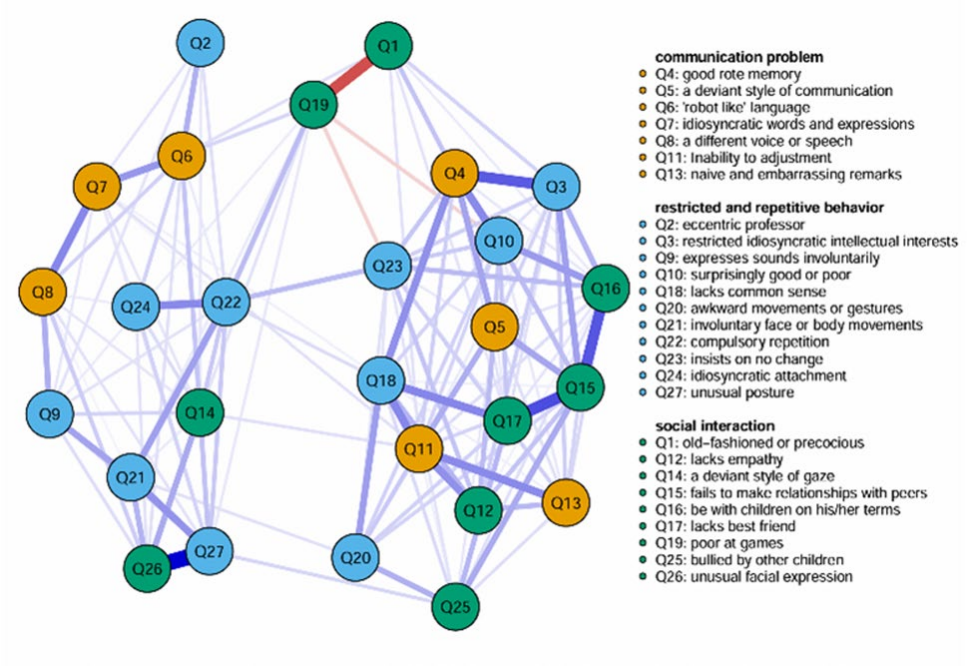
Estimated ASSQ-ising network. Note: The blue solid lines represent positive edges, the red dashed lines represent negative edges, and the thickness of the line indicates the degree of correlation. Edge weights represent log-odds ratios

**Fig. 2:**
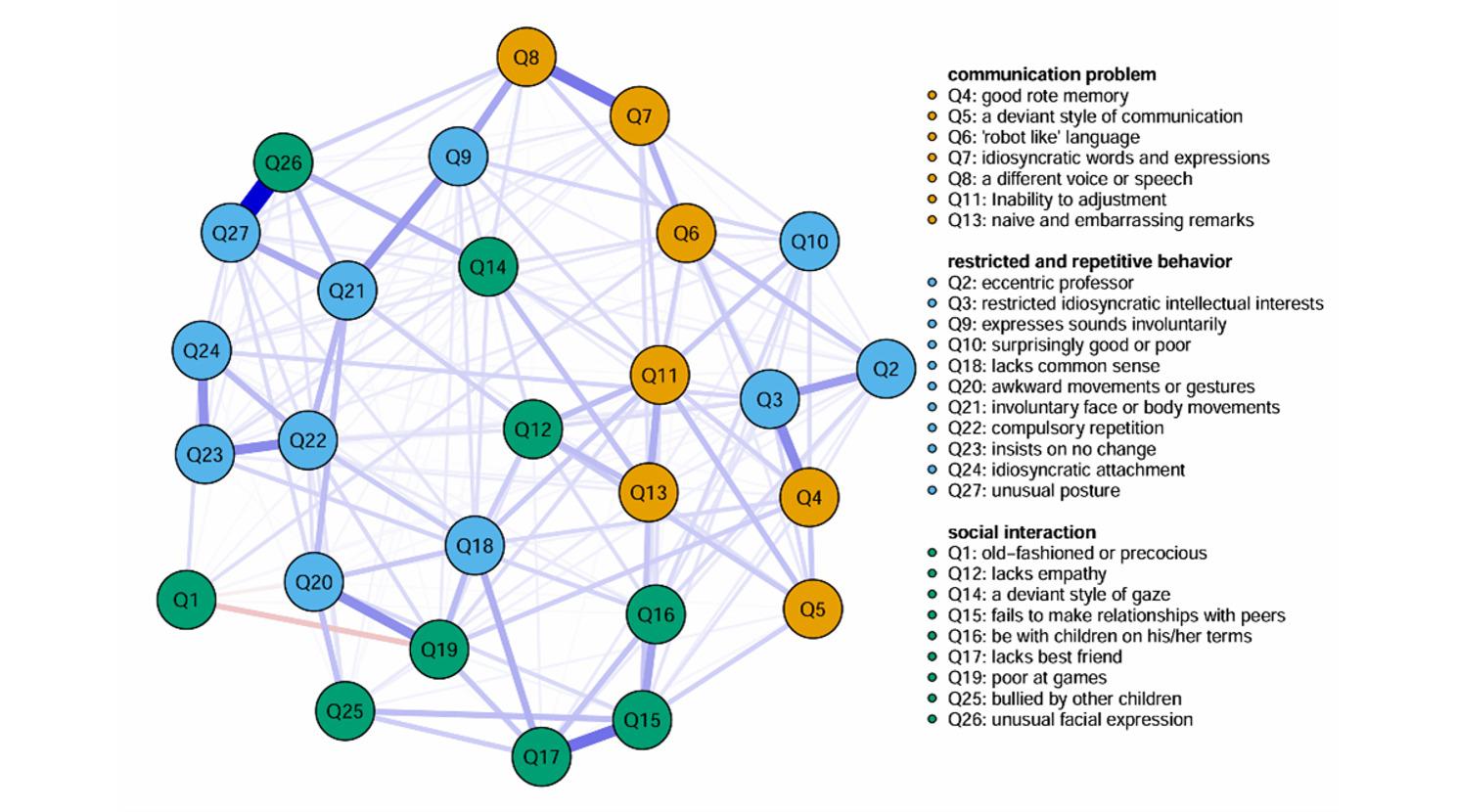
Estimated ASSQ-GGM network. Note: The blue solid line represents a positive correlation, the red dotted line represents a negative correlation, and the thickness of the line indicates the degree of correlation. Edge weights represent partial correlation coefficients

Based on this preprocessing, regularized sparse network estimates were obtained (see Figs. 1 and 2). For an undirected network with 27 nodes, the maximum possible number of edges is 351. After EBIC regularized model selection: The ASSQ-Ising Network retained 134 edges, corresponding to a sparsity of 0.62. The ASSQ-GGM Network retained 192 edges, corresponding to a sparsity of 0.45. It is important to note that the edge weights in the Ising model and the GGM have different statistical interpretations (log-odds ratios and partial correlation coefficients, respectively); therefore, their absolute values should not be directly compared. The strongest pairwise connections in each network were as follows: In the ASSQ-Ising Network (edge weights as log-odds ratios): Q26–Q27 (2.249), Q15–Q17 (1.571), Q1-Q19 (-1.555). In the ASSQ-GGM Network (edge weights as partial correlation coefficients): Q26–Q27 (0.485), Q15–Q17 (0.271), Q7–Q8 (0.260).

#### Centrality estimates of the ASSQ network

**Fig. 3:**
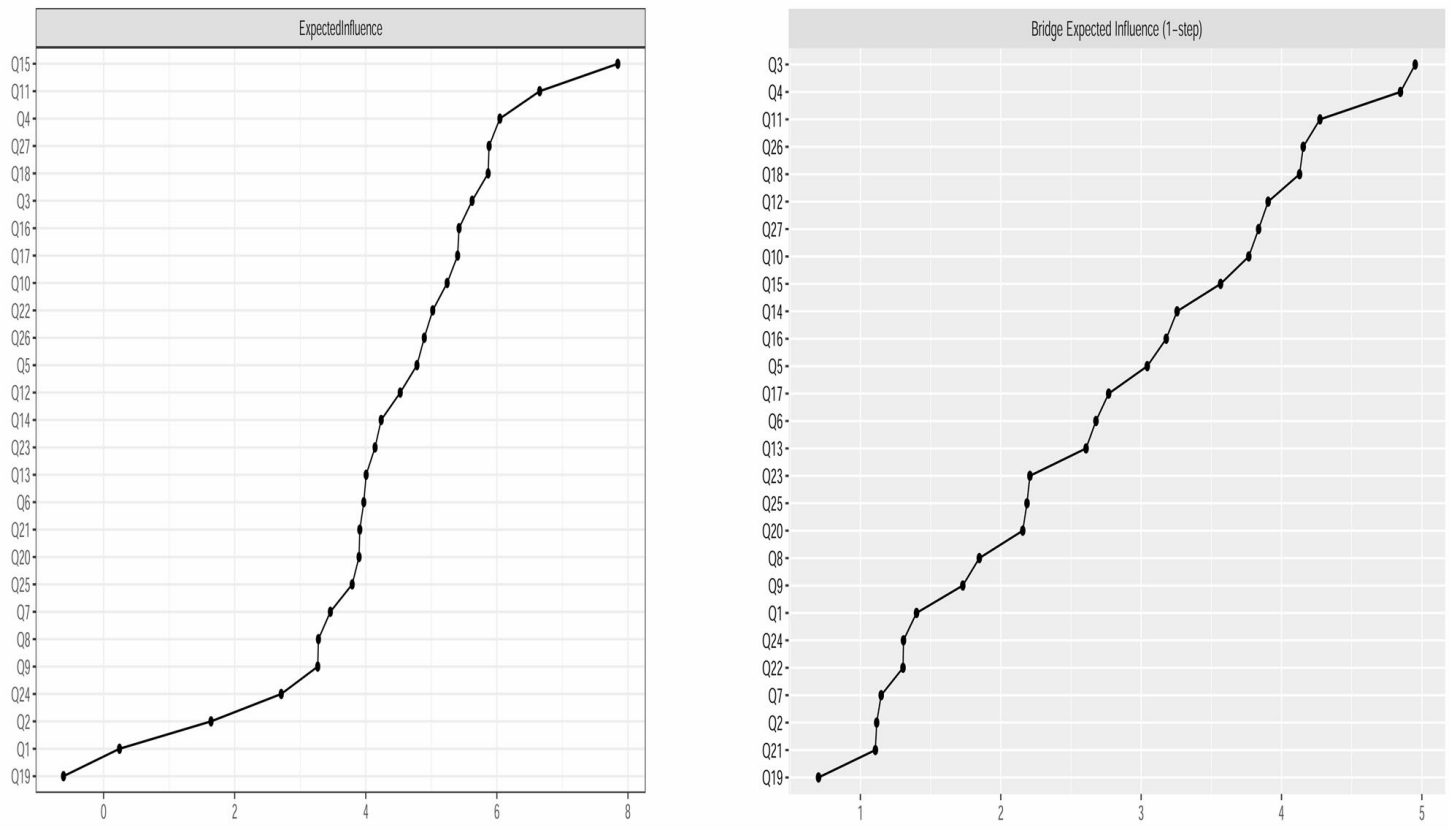
EI (left) and bEI (right) for the ASSQ-ising network. Note: The X-axis indicates the magnitude of the EI value (left) or bEI value (right). The Y-axis lists the corresponding nodes. The order of nodes from top to bottom corresponds to the descending order of their respective values

**Fig. 4:**
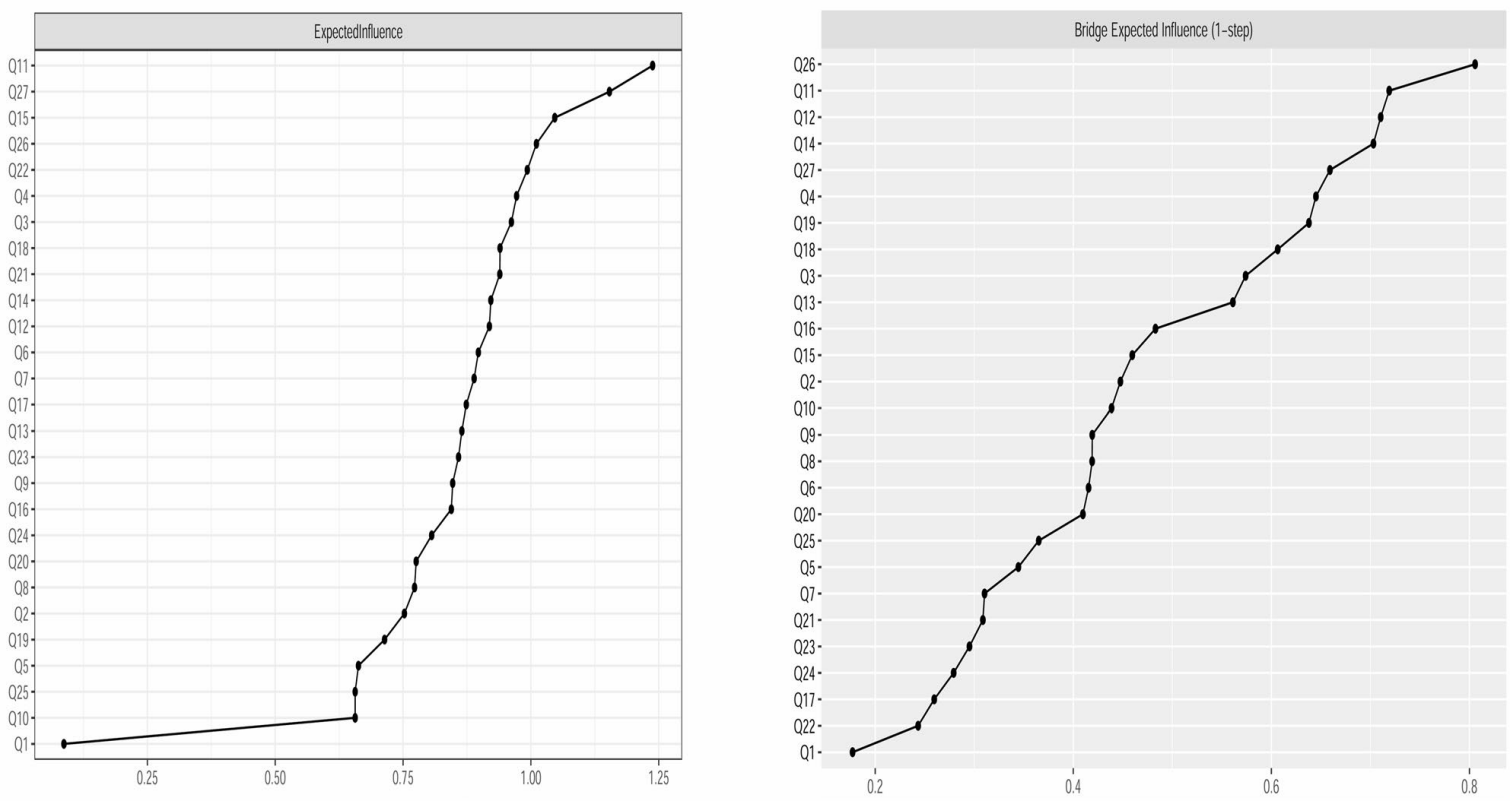
EI (left) and bEI (right) for the ASSQ-GGM network. Note: The X-axis indicates the magnitude of the EI value (left) or bEI value (right). The Y-axis lists the corresponding nodes. The order of nodes from top to bottom corresponds to the descending order of their respective values

Figures 3 and 4 display the centrality metrics, including Expected Influence (EI) and bridge Expected Influence (bEI), for the ASSQ-Ising network and ASSQ-GGM network, respectively. The specific values and their corresponding 95% confidence intervals (CIs) are detailed in Supplementary Table S2.

Centrality analysis revealed the following for the ASSQ-Ising network: The central nodes (highest EI) were Q15 (fails to make relationships with peers, EI = 7.845), Q11 (inability to adjust, EI = 6.653), and Q4 (good rote memory, EI = 6.046). The bridge nodes (highest bEI) were Q3 (restricted idiosyncratic intellectual interests, bEI = 4.953), Q4 (good rote memory, bEI = 4.847), and Q11 (inability to adjust, bEI = 4.274). Given the potential for items with high prevalence in the Ising model to exhibit spuriously high centrality (pseudo-core) due to low thresholds, we conducted correlation analyses between each item’s positive endorsement rate (proportion coded “1”) and its EI and bEI values to examine this potential bias (see Supplementary Table S2 for details). A significant linear effect of item prevalence on centrality metrics was found (EI: *r* = 0.563, *p* = 0.002; bEI: *r* = 0.537, *p*=0.004 ), suggesting that the centrality estimates of the network may be influenced by item prevalence and thus have limited robustness. 

For the ASSQ-GGM network, whose centrality metrics are based on partial correlation coefficients and are therefore not subject to the threshold bias related to item prevalence, the central nodes were Q11 (inability to adjust, EI = 1.238), Q27 (unusual posture, EI = 1.154), and Q15 (fails to make relationships with peers, EI = 1.047). The bridge nodes were Q26 (unusual facial expression, bEI = 0.806), Q11 (inability to adjust, bEI = 0.719), and Q12 (lacks empathy, bEI = 0.710).

#### Stability estimation of the ASSQ network

**Fig. 5:**
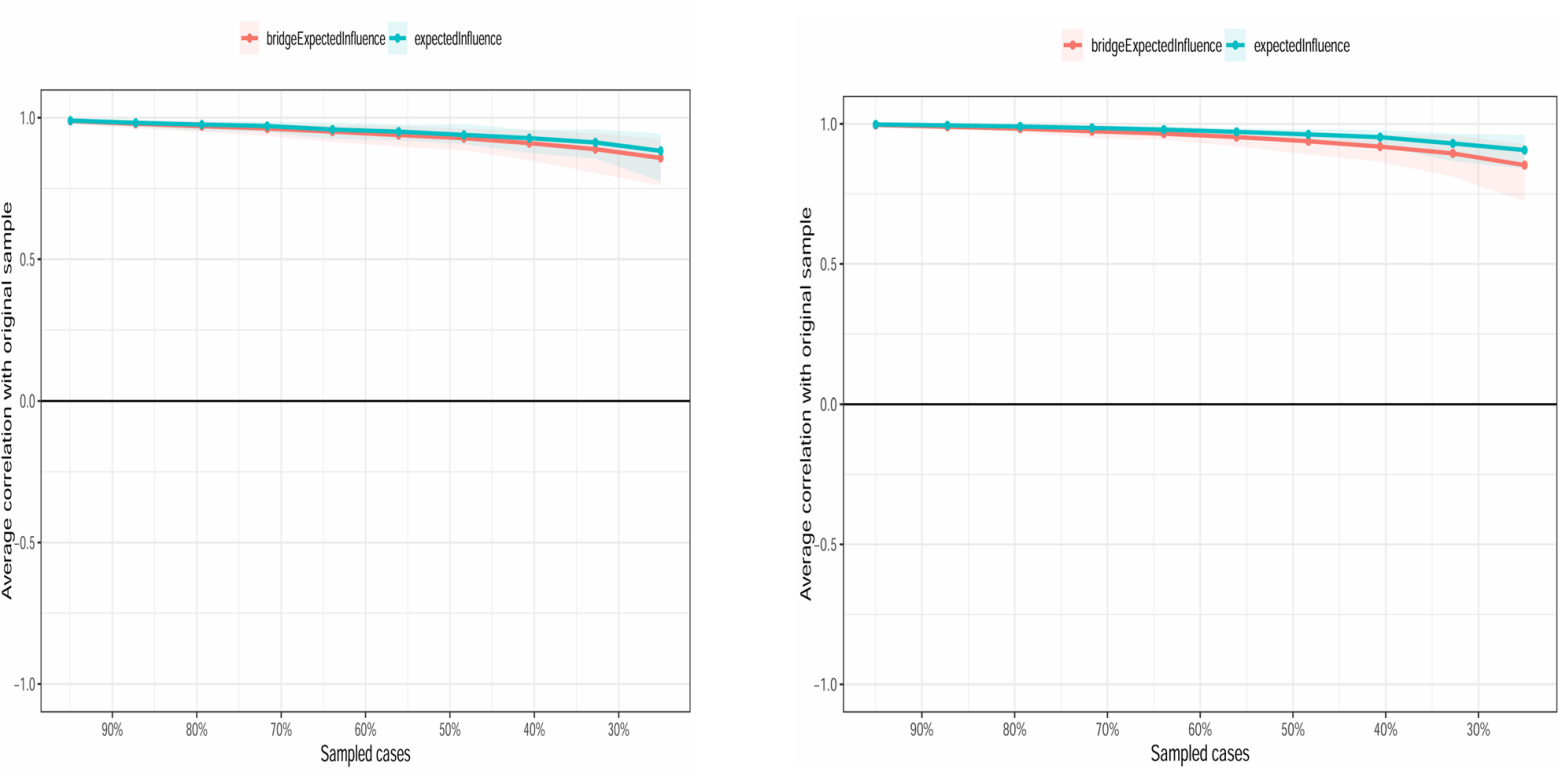
Stability estimation of the ASSQ-Ising network (left) and ASSQ-GGM network (right). Note: The X-axis represents the percentage of cases using the original sample. The Y-axis displays the average correlation between the centrality indices of the original network and those of the re-estimated network

Stability tests were conducted on the Expected Influence (EI) and Bridge Expected Influence (bEI) using the Bootstrap sampling method. The network stability and accuracy assessment results indicated that the correlation stability (CS) coefficients for both EI and bEI in the ASSQ-Ising network and the ASSQ-GGM network were approximately 0.75 (stability assessments for both networks are shown in Fig. 5; difference tests for centrality metrics are presented in Supplementary Figures S1 and S2). All CS coefficients exceeded the recommended threshold of 0.50, indicating good stability in the centrality ranking for both networks. Sensitivity analysis results under different gamma parameters (0, 0.25, 0.5) are provided as supplementary material in Table S3*.*

Furthermore, 95% confidence intervals (CIs) for the edge weights in both networks were calculated using nonparametric Bootstrap (1000 iterations) (see *Figure S3*). The CIs for the vast majority of edges were relatively narrow, suggesting generally high estimation precision for the edge weights. For further quantitative assessment, we defined an edge weight estimate as “acceptable” if the width of its confidence interval (CI_Width) was not greater than half of its absolute edge weight (i.e., CI_Width ≤ |Weight| × 0.5). Supplementary Table S4 summarizes the weights and their confidence intervals for the 10 edges with the highest (strongest) and the 10 edges with the lowest estimated weights in each network. The results showed that in the ASSQ-Ising network, both the strongest edges Q26–Q27 and Q1–Q19 met this “acceptable” criterion; in the ASSQ-GGM network, both the strongest edges Q26–Q27 and Q15–Q17 met the criterion. Difference tests for the edge weights of the two networks are shown in Supplementary Figure S4. Regarding statistical significance: among the 134 edges present in the ASSQ-Ising network, 37 edges (28%) had 95% Bootstrap CIs that did not include zero, indicating these connections were statistically significantly non-zero. Among the 192 edges present in the ASSQ-GGM network, 68 edges (35%) had CIs that did not include zero, representing significantly non-zero edges.

### Model selection

Based on predefined model comparison criteria, a systematic evaluation of the two candidate networks was conducted. Regarding the stability of centrality metrics, the correlation stability (CS) coefficients for both EI and bEI were greater than 0.5 for both the ASSQ-Ising network and the ASSQ-GGM network (0.75 for both), indicating good stability for both. In terms of the estimation precision of edge weights, the Bootstrap confidence intervals for edge weights were generally narrow for both networks. However, examination of the 10 edges with the highest weights revealed that in the ASSQ-Ising network, the two strongest edges (Q26-Q27 and Q1–Q19) met the “acceptable” precision criterion (as defined previously), while in the ASSQ-GGM network, two of the strongest edges (Q26-Q27, Q15-Q17) also satisfied this criterion. Regarding the proportion of statistically significant edges, the ASSQ-GGM network had a higher percentage of edges whose confidence intervals did not include zero (35%) compared to the ASSQ-Ising network (28%). Overall, both the ASSQ-GGM and ASSQ-Ising networks exhibited relatively stable characteristics, with comparable levels of centrality stability and precision in estimating key edges. However, differences were observed in other aspects: the ASSQ-GGM network had a higher proportion of statistically significant edges. Furthermore, the interpretation of network stability in the ASSQ-Ising network was limited to some extent by the correlation between high-prevalence items and their expected influence (EI) and bridge expected influence (bEI) values. Therefore, the ASSQ-GGM network was selected as the final model for this study. Based on this selection, all subsequent network structure comparison tests (e.g., subgroup analyses) and discussions of centrality and bridge centrality metrics will be based on the results from this network.

### Network comparison of the ASSQ network

To examine the potential effects of sex and age on the ASSQ-GGM network structure, Network Comparison Tests (NCT) were performed. Sex Comparison: No significant differences were found between males (*N* = 715) and females (*N* = 280) in global strength (S = 0.46, *p* = 0.63) or overall network structure (M = 0.23, *p* = 0.29). At the edge level, after applying Benjamini-Hochberg (BH) correction for multiple comparisons, no edges showed statistically significant differences in weight between sexes. Age Comparison: Using 12 years as a cutoff, participants were divided into a child group (7–11 years, *N* = 521) and an adolescent group (12–16 years, *N* = 474). No significant differences were found between these groups in global strength (S = 0.17, *p* = 0.84) or overall network structure (M = 0.24, *p* = 0.15). Edge-level analysis with BH correction similarly revealed no significant differences.

It is important to note that when attempting to compute network parameters for each subgroup separately, we encountered limitations: 1. Sample Size Constraints: For a network analysis with 27 variables, the recommended minimum sample size is approximately 540 (20 × N) [12]. The female (*N* = 280), child (*N* = 521), and adolescent (*N* = 474) subsamples in this study did not meet this criterion. 2.Technical Issues: Due to the relatively insufficient sample sizes, the empirical correlation matrices for these subgroups were non-positive definite (encountering ‘S’ is not positive definite error), indicating that the sample sizes were inadequate for stable network estimation in these groups. Consequently: The global strength for the male network was 13.464 (95% CI = [12.946, 19.625]). The point estimate for global strength in the female network was 13.007, but stable confidence intervals could not be calculated. The point estimates for global strength in the child and adolescent networks were 13.060 and 12.888, respectively, and reliable confidence intervals were similarly unavailable.

Therefore, although the NCT found no significant differences, the possibility of subtle sex-specific or age-specific network patterns cannot be ruled out due to the smaller and imbalanced female sample size. Results for the child and adolescent groups, despite having similar sample sizes to each other, should be interpreted with caution as they also fell below the recommended minimum sample size. The lack of significance in edge-level difference analyses may be attributed to sample imbalance and multiple comparison correction, and potential differences warrant further investigation in larger samples.

## Discussion

This study employed network analysis to identify central and bridge nodes within the ASSQ network of children and adolescents with ASD. By comparing two distinct statistical approaches, the ASSQ-GGM model, which demonstrated stronger centrality, greater edge weight stability, and higher accuracy, was selected. The central nodes in this network were: “inability to adjust”, “unusual posture” and “fails to make relationships with peers”. The bridge nodes were: “unusual facial expression”, “inability to adjust” and “lacks empathy”.

Since existing research on the ASSQ lacks detailed reports of factor loadings, the network analysis results from this study can be cautiously compared with the factor structure of the classic Autism Spectrum Quotient (AQ) scale [32]. The validation study of the AQ-Child established four core dimensions through factor analysis: “mind-reading,” “attention to detail,” “social skills,” and “imagination,” and identified items with high loadings on these factors. In a specific correspondence to this: the central node “inability to adjust” in our network conceptually aligns well with the item with the highest loading (0.663) on the “mind-reading” dimension (“Keeps going on and on about the same thing”). Another central node, “fails to make relationships with peers,” corresponds to the item with the highest loading (-0.777) on the “social skills” dimension (“enjoy social occasions”). These two independent methodologies (cross-sectional network analysis and factor analysis) yielded mutually corroborating results in identifying core items related to communication problems and social interaction.

“Inability to adjustment to fit social contexts or the needs of different listeners” emerged as the primary central node in the ASSQ network. This finding may be partly explained from the perspective of theory of mind, which posits that individuals with ASD have impairments in understanding their own or others’ mental states [33]. While some children with ASD can generate thoughts, beliefs, and intentions in theory-of-mind tasks, deficits in their social skills hinder the application of these abilities in real-world social situations [34, 35]. Although this study did not directly measure theory of mind, and this explanation remains theoretical, the identification of this core node provides an empirical focus for future research to directly examine the specific link between social adjustment difficulties and mental state attribution deficits in ASD. Some researchers have found that children with ASD experience self-regulation difficulties, and challenges with switching can predict their level of adaptive functioning [36]. Individuals with ASD who have better self-regulation are more likely to achieve closer friendships and higher friendship quality [37]. Thus, “difficulty adjusting” as a central node may also reflect the pervasive difficulty individuals with ASD face in regulating their behavior, emotions, and cognitive responses within rapidly changing social environments.

“Unusual posture” was the second central node identified in the ASSQ network. This finding resonates with research based on other instruments. For example, a network analysis using ADOS scores found that in Module 1, non-verbal communication items such as “gestures” and “abnormal eye contact” had high centrality [13]. Multiple studies suggest that motor abnormalities hold a significant position in ASD: atypical body movement patterns in infancy can serve as a potential early biomarker for ASD [38]; some research indicates that individuals with ASD, particularly preschool-aged girls, frequently have comorbid developmental coordination disorder [39]; their postural control systems often exhibit developmental immaturity, and their gait and postural control patterns are unique [40]. At the neural level, cerebellar dysfunction has also been implicated in the motor and non-motor symptoms of ASD [41]. In summary, the cross-sectional network analysis of the parent-reported ASSQ in this study identified “abnormal posture” as central, suggesting that postural and movement-related abnormalities may be a notable behavioral feature in the clinical presentation of ASD. “Wishes to be sociable but fails to make relationships with peers” was a central node in the ASSQ network. This finding captures the common disconnection between “social motivation and social ability” observed in individuals with ASD. Previous research has extensively explored the complex composition of social difficulties in ASD from different angles. For instance, a network analysis study suggested that alexithymia (difficulty identifying and describing emotions) might be a key central feature within a psychological structure involving autistic traits, interoception, and empathy [42]. Furthermore, substantial research confirms that social communication difficulties in individuals with ASD are closely related to their mental health risks; for example, they significantly increase the likelihood of developing depression [43] and experiencing bullying [44]. Together, this literature suggests that the ASSQ item “fails to make relationships” may be linked to multi-faceted cognitive and affective processing difficulties within the social phenotype of ASD and has important implications for long-term social adaptation and mental health. Bridge symptoms refer to nodes that have strong connections to other dimensions of the ASSQ (social interaction, communication problems, restricted repetitive behaviors). Identifying such nodes can help understand potential pathways for symptom transmission across dimensions and may reveal key intervention targets. In this study, “unusual facial expression” was the first identified bridge node. Extensive research indicates that individuals with ASD have difficulties with both the production (expression) and comprehension (recognition) of facial expressions: the quality of their facial expressions is significantly lower and their expressiveness is less accurate compared to typically developing individuals [45]; moreover, the quality of their facial expressions is positively correlated with their social communication abilities [46]. These behavioral-level deficits may stem from their unique neural processing mechanisms. Neuroimaging studies suggest that abnormal facial expression processing in ASD involves multiple levels, including abnormal primary visual processing of faces, atypical activation patterns in key “social brain” nodes during expression recognition, and disrupted functional connectivity within and between these brain regions [47]. Therefore, the bridging position of the “abnormal facial expression” item in the ASSQ network may reflect its dual characteristics of connecting different symptom domains at the behavioral level and involving core pathways of social information processing at the neural level. 

“Inability to adjustment to fit social contexts or the needs of different listeners” was identified as the second important bridge node in the network, indicating that it conceptually and statistically connects multiple typical symptom domains in children with ASD, including restricted repetitive behaviors, social interaction, and communication difficulties. Research suggests that difficulties in adjusting to social contexts or listener needs may be related to deficits in social and cognitive mechanisms, which in turn may be closely associated with the developmental level of an individual’s social skills and cognitive functions [48].

“Lacks empathy” was identified as the third important bridge node in the network. Studies have found that individuals with ASD exhibit empathy deficits [49], and adolescents with higher autistic traits demonstrate significantly lower empathic ability compared to those with lower autistic traits [50]. Empathy is closely related to social and communication difficulties. Researchers have noted that individuals with ASD show deficits in the accuracy of empathy for anger and exhibit lower perspective-taking and empathic concern scores on the Interpersonal Reactivity Index [51]. This suggests that, as a bridge node, "lack of empathy" may connect the typical difficulties experienced by individuals with ASD in social and communication domains, reflecting the intrinsic associations among these symptom dimensions within the ASSQ network structure. 

Based on the network comparison analysis of the available sample, this study did not find statistically significant differences related to sex or age. This result is inconsistent with some existing research and requires cautious interpretation considering the sample characteristics. Regarding sex: This analysis did not detect significant sex differences. However, it is noteworthy that previous studies, using different instruments (e.g., ADOS modules) [13] or focusing on specific functional domains (e.g., social-emotional) [14], have reported the existence of sex differences, with ASD males showing higher density in social-emotional networks or females showing greater network strength for ADOS Module 1 items than males. The inconsistency between our findings and these reports may partly stem from differences in the item properties of the scales used (ASSQ), the analytical dimensions, and importantly, the limitation of the relatively smaller female subsample size (*N* = 280) in this study. For a network analysis involving 27 items, the current female sample size may have been insufficient to fully capture potentially subtle sex-specific network patterns. Regarding age: This study divided the sample into child (7–11 years) and adolescent (12–16 years) groups, and the Network Comparison Test found no significant differences. This aligns with the study by Zhang et al. [52], which also indicated no significant structural differences between child and adolescent networks. However, it is also important to note that while the sample sizes for the child (*N* = 521) and adolescent (*N* = 474) groups in this study were comparable, neither met the recommended sufficient sample size based on the number of variables. This may have limited the statistical power to detect potential subtle age-related differences.

## Conclusion

Based on the analysis of the ASSQ symptom network in children and adolescents with ASD, this study identified three central nodes: “inability to adjust”, “unusual posture” and “fails to make relationships with peers”. It also identified three bridge nodes: “unusual facial expression”, “inability to adjust” and “lacks empathy.” Notably, “inability to adjust” exhibited both core and bridge properties, occupying a hub position within the network. These findings indicate that the ASD behavioral features assessed by the ASSQ are interconnected through specific nodes. Core nodes may reflect common difficulties experienced by individuals with ASD in social adaptation and flexibility, non-verbal behavioral patterns, and the tension between social motivation and actual social connectedness. Bridge nodes, in turn, suggest that non-verbal expression and empathic ability may play important roles in connecting different behavioral domains. Based on the current sample, the network comparison analysis did not reveal significant differences in network structure between sex or age groups. However, this result may be limited by subsample sizes and statistical power and requires further validation in future studies. In summary, this study preliminarily delineates the internal associative structure among ASSQ symptoms and identifies a set of potential central and bridge symptoms. These nodes can provide focal points for subsequent research—for example, investigating whether interventions targeting such hub features might yield benefits across broader functional domains. It is crucial to emphasize that the conclusions of this study are derived from cross-sectional data and a specific measurement tool. The identified network structure and its hub nodes await testing in different samples and longitudinal studies.

### Limitations and perspectives

First, this study is based on cross-sectional data. The revealed symptom association network cannot infer causal relationships or the dynamic evolution of symptoms over time. Therefore, any intervention hypotheses proposed based on central or bridge nodes are exploratory and require testing for causal efficacy through longitudinal studies or experimental designs (directed acyclic graphs are also a potential consideration). Second, while the network comparison analysis did not find significant sex or age differences, this negative result may be influenced by insufficient subsample sizes. Specifically, the female subsample (*N* = 280) did not meet the recommended sample size guidelines for stable network estimation, potentially limiting statistical power and the stability of parameter estimates. Future research is needed to validate the generalizability of the network structure in larger and more balanced samples. Third, there was substantial missing data for a key covariate. A significant limitation is the high rate of missing data for intellectual level (only 37.79% of the sample had complete data). Given that intellectual functioning is a core covariate influencing the clinical presentation of ASD, its incomplete data may have restricted a more nuanced analysis of symptom network heterogeneity and could affect the generalizability of the findings. Additionally, this study did not account for other potentially relevant factors such as genetic and family background information, or comorbid conditions like developmental coordination disorder. Fourth, this study relied entirely on parent-reported ASSQ data. Consequently, the presented network structure reflects behavioral associations from the parent’s observational perspective, rather than the individual’s internal psychopathological processes. Furthermore, the scale itself may possess measurement properties—such as item overlap, factor instability, and informant effects—that could influence the precision of the network structure. In summary, this study provides a perspective on the internal associations among ASD behavioral features as assessed by the ASSQ and identifies a set of potentially key symptom targets. However, all conclusions must be understood within the framework of the methodological limitations outlined above and await further validation in different samples, with multi-informant data (e.g., teacher reports, clinical observation), and through longitudinal designs.

## Electronic Supplementary Material

Below is the link to the electronic supplementary material.

Supplementary Material 1

## Data Availability

All data generated or analyzed during this study are included in this published article.
