# Supplementary material for: Exploring autism spectrum profiles via network analysis of parent-reported ASSQ patterns
Source: BMC Psychiatry. 2026 Mar 5;26:261. doi: 10.1186/s12888-026-07935-z (PMC13023168; doi:10.1186/s12888-026-07935-z)
Supplement: Supplementary file 1 — Supplementary Material 1 [file 12888_2026_7935_MOESM1_ESM.docx]

**Supplementary material**

*Table* S*1* Abbreviations for entries in the Autism Spectrum Screening Questionnaire

| items | | Abbreviation |
| --- | --- | --- |
| Q1 | is old-fashioned or precocious | old-fashioned or precocious |
| Q2 | is regarded as an “eccentric professor”by the other children | eccentric professor |
| Q3 | lives somewhat in a world of his/her own with restricted idiosyncratic intellectual interests | restricted idiosyncratic intellectual interests |
| Q4 | accumulates facts on certain subjects (good rote memory) but does not really understand the meaning | good rote memory |
| Q5 | has a literal understanding of ambiguous and metaphorical language has a deviant style of communication | a deviant style of communication |
| Q6 | with a formal, fussy, old-fashioned or “robot like” language | “robot like” language |
| Q7 | invents idiosyncratic words and expressions | idiosyncratic words and expressions |
| Q8 | has a different voice or speech | a different voice or speech |
| Q9 | expresses sounds involuntarily：clears throat, grunts, smacks, cries or screams | expresses sounds involuntarily |
| Q10 | is surprisingly good at some things and surprisingly poor at others uses language freely but fails to make | surprisingly good or poor |
| Q11 | Inability to adjustment to fit social contexts or the needs of different listeners | inability to adjust |
| Q12 | lacks empathy | lacks empathy |
| Q13 | makes naive and embarrassing remarks | naive and embarrassing remarks |
| Q14 | has a deviant style of gaze | a deviant style of gaze |
| Q15 | wishes to be sociable but fails to make relationships with peers | fails to make relationships with peers |
| Q16 | can be with other children but only on his/her terms | be with children on his/her terms |
| Q17 | lacks best friend | lacks best friend |
| Q18 | lacks common sense | lacks common sense |
| Q19 | is poor at games: no idea of cooperating in a team, scores “own goals” | poor at games |
| Q20 | has clumsy, ill coordinated, ungainly, awkward movements or gestures | awkward movements or gestures |
| Q21 | has involuntary face or body movements has difficulties in completing simple daily | involuntary face or body movements |
| Q22 | activities because of compulsory repetition of certain actions or thoughts | compulsory repetition |
| Q23 | has special routines: insists on no change | insists on no change |
| Q24 | shows idiosyncratic attachment to objects | idiosyncratic attachment |
| Q25 | is bullied by other children | bullied by other children |
| Q26 | has markedly unusual facial expression | unusual facial expression |
| Q27 | has markedly unusual posture | unusual posture |

*Table S2* Centrality Metrics of Network Models and Item Positive Proportion in the Ising Model

| Code |  | | ASSQ-Ising Network | | |  | ASSQ-GGM Network | | | |
| --- | --- | --- | --- | --- | --- | --- | --- | --- | --- | --- |
|  | N(%) | EI | EI [95%CI] | bEI | bEI [95%CI] |  | EI | EI [95%CI] | bEI | bEI [95%CI] |
| Q1 | 251（25.2%） | 0.242 | [-0.656, 0.858] | 1.400 | [0.644, 1.977] |  | 0.087 | [-0.008, 0.201] | 0.177 | [0.083, 0.301] |
| Q2 | 439（44.1%） | 1.639 | [1.095, 2.680] | 1.116 | [0.624, 1.882] |  | 0.753 | [0.638, 0.867] | 0.448 | [0.324, 0.582] |
| Q3 | 704（70.8%） | 5.622 | [4.876, 7.075] | 4.953 | [3.991, 6.042] |  | 0.962 | [0.844, 1.078] | 0.574 | [0.466, 0.716] |
| Q4 | 685（68.8%） | 6.046 | [5.329, 7.542] | 4.847 | [4.039, 6.076] |  | 0.972 | [0.865, 1.079] | 0.645 | [0.512, 0.771] |
| Q5 | 656（65.9%） | 4.781 | [4.005, 5.919] | 3.044 | [2.179, 4.128] |  | 0.663 | [0.569, 0.763] | 0.345 | [0.239, 0.466] |
| Q6 | 408（41.0%） | 3.970 | [2.670, 4.667] | 2.678 | [1.430, 3.144] |  | 0.897 | [0.758, 1.004] | 0.415 | [0.282, 0.570] |
| Q7 | 344（34.6%） | 3.460 | [2.638, 4.357] | 1.148 | [0.567, 1.835] |  | 0.889 | [0.779, 0.994] | 0.310 | [0.220, 0.462] |
| Q8 | 330（33.2%） | 3.278 | [2.816, 4.484] | 1.847 | [1.451, 2.957] |  | 0.772 | [0.677,0.901] | 0.419 | [0.335, 0.557] |
| Q9 | 415（41.7%） | 3.267 | [2.371, 3.898] | 1.730 | [0.969, 2.319] |  | 0.847 | [0.735, 0.938] | 0.419 | [0.305, 0.538] |
| Q10 | 797（19.9%） | 5.241 | [4.177, 6.044] | 3.767 | [2.767, 4.765] |  | 0.656 | [0.565, 0.756] | 0.439 | [0.342, 0.562] |
| Q11 | 641（64.4%） | 6.653 | [5.335, 7.952] | 4.274 | [3.103, 5.366] |  | 1.238 | [1.128, 1.333] | 0.719 | [0.589, 0.849] |
| Q12 | 629（63.2%） | 4.526 | [3.856, 5.340] | 3.905 | [3.083, 4.788] |  | 0.919 | [0.804, 1.017] | 0.710 | [0.587, 0.838] |
| Q13 | 545（54.8%） | 4.006 | [3.504, 5.475] | 2.608 | [1.937, 3.810] |  | 0.865 | [0.751, 0.972] | 0.561 | [0.429, 0.681] |
| Q14 | 380（38.2%） | 4.235 | [3.113, 4.979] | 3.256 | [2.285, 3.980] |  | 0.922 | [0.823, 1.050] | 0.703 | [0.546, 0.835] |
| Q15 | 786（79.0%） | 7.845 | [6.516, 8.704] | 3.566 | [2.437, 4.409] |  | 1.047 | [0.915, 1.149] | 0.460 | [0.350, 0.564] |
| Q16 | 714（71.8%） | 5.423 | [4.535, 6.402] | 3.179 | [2.433, 4.183] |  | 0.844 | [0.750, 0.964] | 0.483 | [0.364, 0.604] |
| Q17 | 709（71.3%） | 5.401 | [4.204, 6.052] | 2.769 | [1.761, 3.448] |  | 0.874 | [0.758, 0.976] | 0.260 | [0.189, 0.383] |
| Q18 | 623（62.6%） | 5.867 | [4.652, 6.806] | 4.129 | [3.256, 4.941] |  | 0.940 | [0.845, 1.034] | 0.606 | [0.497, 0.744] |
| Q19 | 574（57.7%） | -0.612 | [-1.663, 0.131] | 0.702 | [-0.240, 1.432] |  | 0.714 | [0.612, 0.821] | 0.638 | [0.534, 0.734] |
| Q20 | 540（54.3%） | 3.897 | [3.246, 5.283] | 2.157 | [1.499, 3.248] |  | 0.776 | [0.671, 0.885] | 0.410 | [0.296, 0.518] |
| Q21 | 457（45.9%） | 3.909 | [2.973, 4.852] | 1.107 | [0.485, 1.926] |  | 0.939 | [0.852, 1.046] | 0.309 | [0.216, 0.453] |
| Q22 | 451（45.3%） | 5.021 | [3.686, 5.751] | 1.304 | [0.647, 2.055] |  | 0.993 | [0.886, 1.111] | 0.243 | [0.145, 0.394] |
| Q23 | 582（58.5%） | 4.142 | [3.155, 5.190] | 2.208 | [1.278, 3.119] |  | 0.859 | [0.762, 0.974] | 0.295 | [0.179, 0.418] |
| Q24 | 370（37.2%） | 2.710 | [2.181, 3.871] | 1.307 | [0.587, 2.011] |  | 0.806 | [0.680, 0.911] | 0.279 | [0.144, 0.410] |
| Q25 | 535（53.8%） | 3.793 | [2.982, 4.844] | 2.187 | [1.456, 3.282] |  | 0.656 | [0.569, 0.769] | 0.365 | [0.240, 0.483] |
| Q26 | 360（36.2%） | 4.892 | [4.112, 5.857] | 4.155 | [3.394, 5.107] |  | 1.011 | [0.884, 1.099] | 0.806 | [0.694, 0.911] |
| Q27 | 367（36.9%） | 5.881 | [4.522, 7.065] | 3.837 | [2.724, 4.655] |  | 1.154 | [1.040, 1.241] | 0.659 | [0.540, 0.776] |

*Note:* EI = Expected Influence; bEI = bridge Expected Influence. For the Ising model: N (%) indicates the number and percentage of cases for which each node was scored as "1" (symptom present). To examine potential bias due to item prevalence, we calculated the correlation between this positive endorsement rate and the node's centrality. Significant linear associations were found (with EI: r = 0.563, p = 0.002; with bEI: r = 0.537, p = 0.004).

*Table S3* Sensitivity Analysis of Network Stability under Different Gamma Parameters

|  |  | gamma=0 | gamma=0.25 | gamma=0.5 |
| --- | --- | --- | --- | --- |
| ASSQ-Ising network | EI | CS=0.75 | CS=0.75 | CS=0.75 |
|  | bEI | CS=0.75 | CS=0.75 | CS=0.75 |
| ASSQ-GGM network | EI | CS=0.75 | CS=0.75 | CS=0.672 |
|  | bEI | CS=0.75 | CS=0.75 | CS=0.672 |

*Table S4* Summary of Selected Edge Weights with 95% Bootstrap Confidence Intervals

| ASSQ-Ising Network | | | | |  | ASSQ-GGM Network | | | | |
| --- | --- | --- | --- | --- | --- | --- | --- | --- | --- | --- |
| Edge | Weight | CI_Width | CI_Lower | CI_Upper |  | Edge | Weight | CI_Width | CI_Lower | CI_Upper |
| Q26-Q27* | 2.249 | 0.758 | 1.884 | 2.642 |  | Q26-Q27* | 0.485 | 0.130 | 0.416 | 0.546 |
| Q15-Q17* | 1.571 | 0.951 | 1.060 | 2.011 |  | Q15-Q17* | 0.271 | 0.105 | 0.212 | 0.317 |
| Q1-Q19* | -1.555 | 0.640 | -1.830 | -1.190 |  | Q7-Q8* | 0.260 | 0.135 | 0.181 | 0.316 |
| Q15-Q16* | 1.474 | 0.990 | 0.981 | 1.972 |  | Q22-Q23* | 0.221 | 0.120 | 0.154 | 0.274 |
| Q3-Q4* | 1.451 | 0.907 | 1.030 | 1.937 |  | Q3-Q4* | 0.220 | 0.123 | 0.156 | 0.279 |
| Q17-Q18* | 1.050 | 0.926 | 0.497 | 1.422 |  | Q19-Q20* | 0.215 | 0.116 | 0.153 | 0.269 |
| Q11-Q18* | 1.039 | 0.955 | 0.555 | 1.510 |  | Q23-Q24* | 0.210 | 0.122 | 0.146 | 0.267 |
| Q7-Q8* | 1.039 | 0.631 | 0.737 | 1.368 |  | Q9-Q21* | 0.194 | 0.139 | 0.116 | 0.255 |
| Q22-Q24* | 1.028 | 0.631 | 0.690 | 1.320 |  | Q2-Q3* | 0.185 | 0.117 | 0.123 | 0.240 |
| Q4-Q10* | 1.017 | 0.936 | 0.514 | 1.449 |  | Q15-Q16* | 0.174 | 0.113 | 0.114 | 0.227 |
| Q8-Q24 | 0.163 | 0.511 | 0.000 | 0.511 |  | Q18-Q27 | 0.050 | 0.000 | 0.100 | 0.100 |
| Q9-Q19 | 0.162 | 0.455 | 0.000 | 0.455 |  | Q12-Q19 | 0.050 | 0.000 | 0.106 | 0.106 |
| Q5-Q17 | 0.157 | 0.590 | 0.000 | 0.590 |  | Q10-Q15 | 0.049 | 0.000 | 0.100 | 0.100 |
| Q11-Q25 | 0.133 | 0.578 | 0.000 | 0.578 |  | Q3-Q6 | 0.049 | 0.000 | 0.106 | 0.106 |
| Q7-Q13 | 0.127 | 0.527 | 0.000 | 0.527 |  | Q8-Q27 | 0.049 | 0.000 | 0.105 | 0.105 |
| Q15-Q18 | 0.127 | 0.616 | 0.000 | 0.616 |  | Q6-Q24 | 0.045 | 0.000 | 0.093 | 0.093 |
| Q12-Q16 | 0.116 | 0.543 | 0.000 | 0.543 |  | Q14-Q27 | 0.045 | 0.000 | 0.104 | 0.104 |
| Q8-Q21 | 0.098 | 0.430 | 0.000 | 0.430 |  | Q2-Q4 | 0.043 | 0.000 | 0.099 | 0.099 |
| Q7-Q22 | 0.085 | 0.374 | 0.000 | 0.374 |  | Q3-Q11 | 0.043 | 0.000 | 0.095 | 0.095 |
| Q6-Q27 | 0.050 | 0.423 | 0.000 | 0.423 |  | Q16-Q22 | 0.042 | 0.000 | 0.086 | 0.086 |

*Note:*An edge is considered to have "acceptable" estimation precision if it meets the criterion CI_Width ≤ |Weight| × 0.5. In the Ising model, the edge Q26–Q27 and Q1-Q19 meet this criterion. In the GGM model, the edges Q26–Q27 and Q15–Q17 meet this criterion. This table presents the 10 edges with the largest absolute edge weights (strongest edges) and the 10 edges with the widest 95% Bootstrap confidence intervals (i.e., edges with the lowest estimation precision) for each network model. An asterisk (*) beside an edge indicates that its 95% confidence interval does not include zero, meaning the edge weight is statistically significantly non-zero. Edges without an asterisk are not statistically significant.


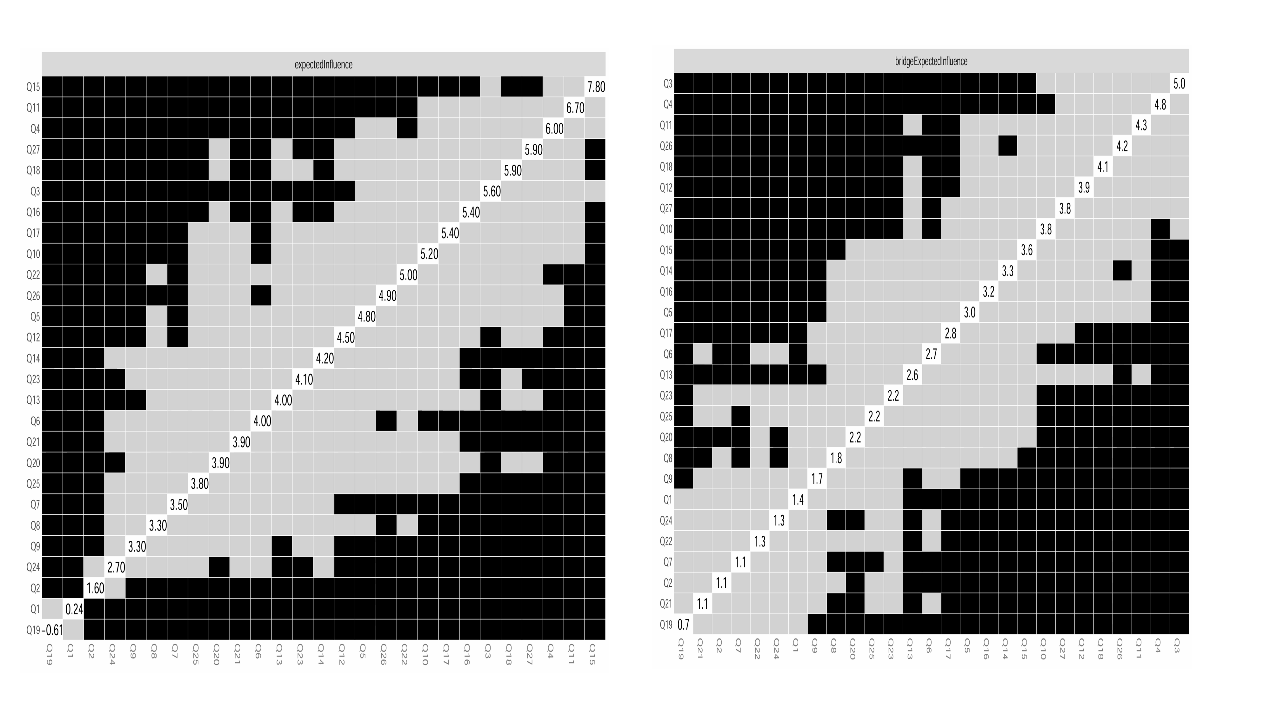


*Note:* In the figure, a black square indicates a statistically significant difference between the Expected Influence (EI) or bridge Expected Influence (bEI) values of the two nodes corresponding to its row and column (based on a 95% confidence interval test from 1000 bootstrap samples; a significant difference at p < 0.05 is determined if the confidence interval for the difference between the two nodes' centrality measures does not include zero). A grey square indicates no significant difference. To identify key nodes in the network from a statistical comparison perspective, we operationally defined the central nodes as the top three nodes with the greatest number of significant differences from other nodes in the EI difference test (Q15, Q11, Q4). Similarly, we defined the bridge nodes as the top three nodes with the greatest number of significant differences from other nodes in the bEI difference test (Q3, Q4, Q11).

*Figure S1*  EI（left）and bEI（right）Difference Test Plot in the ASSQ-Ising Network


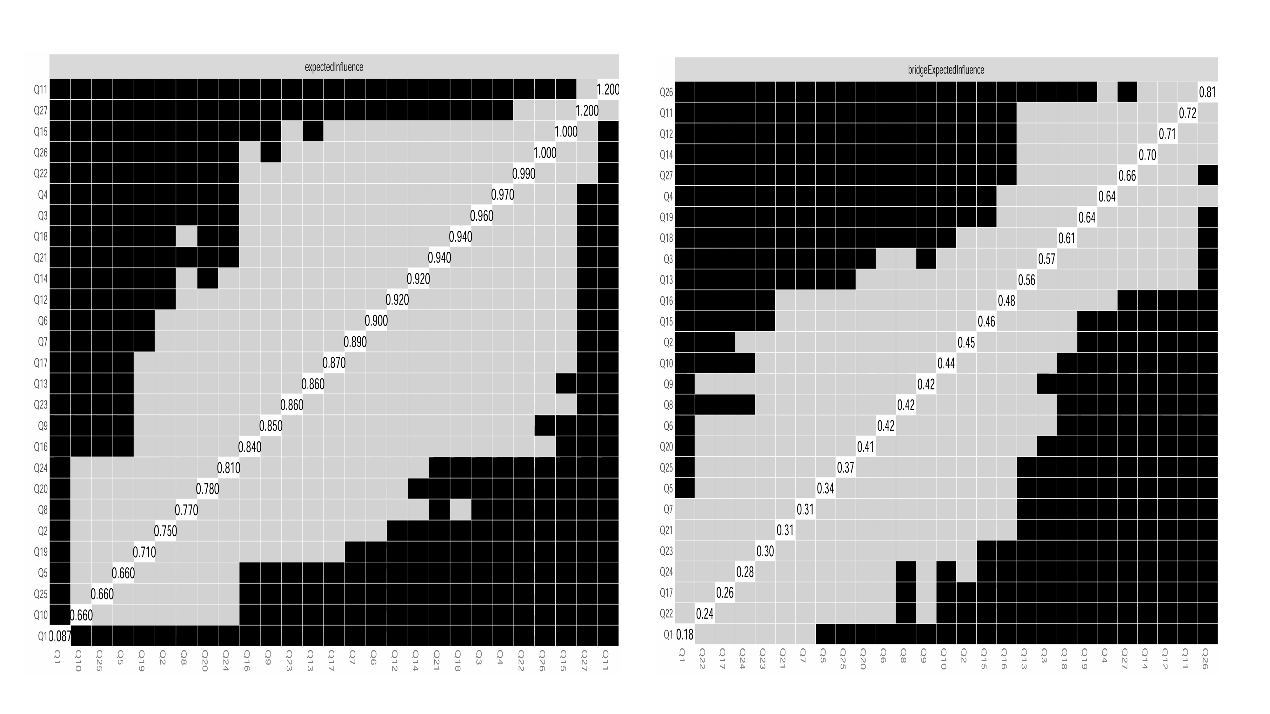


*Note:* In the figure, a black square indicates a statistically significant difference between the Expected Influence (EI) or bridge Expected Influence (bEI) values of the two nodes corresponding to its row and column (based on a 95% confidence interval test from 1000 bootstrap samples; a significant difference at p < 0.05 is determined if the confidence interval for the difference between the two nodes' centrality measures does not include zero). A grey square indicates no significant difference. To identify key nodes in the network from a statistical comparison perspective, we operationally defined the central nodes as the top three nodes with the greatest number of significant differences from other nodes in the EI difference test (Q11, Q27, Q15). Similarly, we defined the bridge nodes as the top three nodes with the greatest number of significant differences from other nodes in the bEI difference test (Q26, Q11, Q12).

*Figure S2* EI（left）and bEI（right）Difference Test Plot in the ASSQ-GGM Network


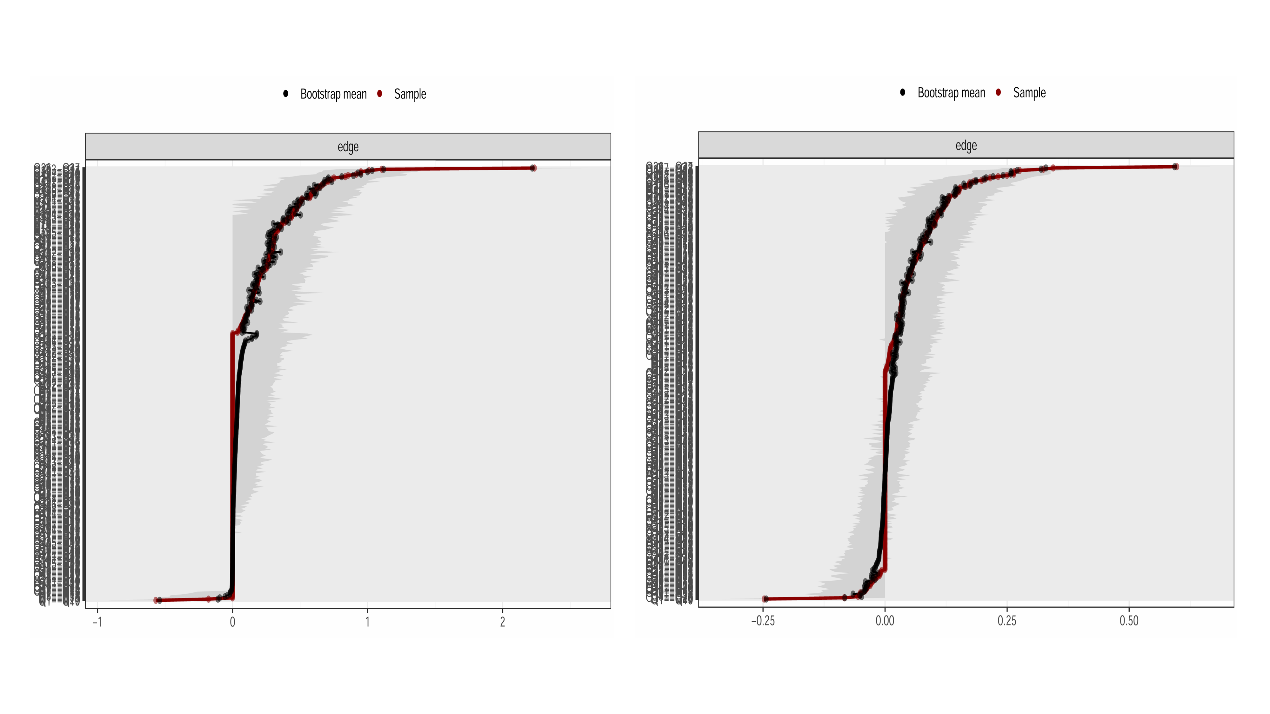
 *Note:* This figure displays the point estimates (black dots) and their corresponding 95% bootstrap confidence intervals (vertical lines) for each edge weight in the network. Narrower confidence intervals indicate higher estimation precision for the respective edge weight. The horizontal gray dashed line represents the reference line of zero weight. If the confidence interval of an edge lies entirely above or below this line (i.e., does not cross it), the edge weight is statistically significantly non-zero (p < 0.05). The vertical axis lists all edges, typically ordered from strongest to weakest (or according to a specific sequence), while the horizontal axis represents the magnitude of the edge weights.

Figure S3 Accuracy Verification of Edges in the ASSQ-Ising Network(left) and ASSQ-GGM Network(right)


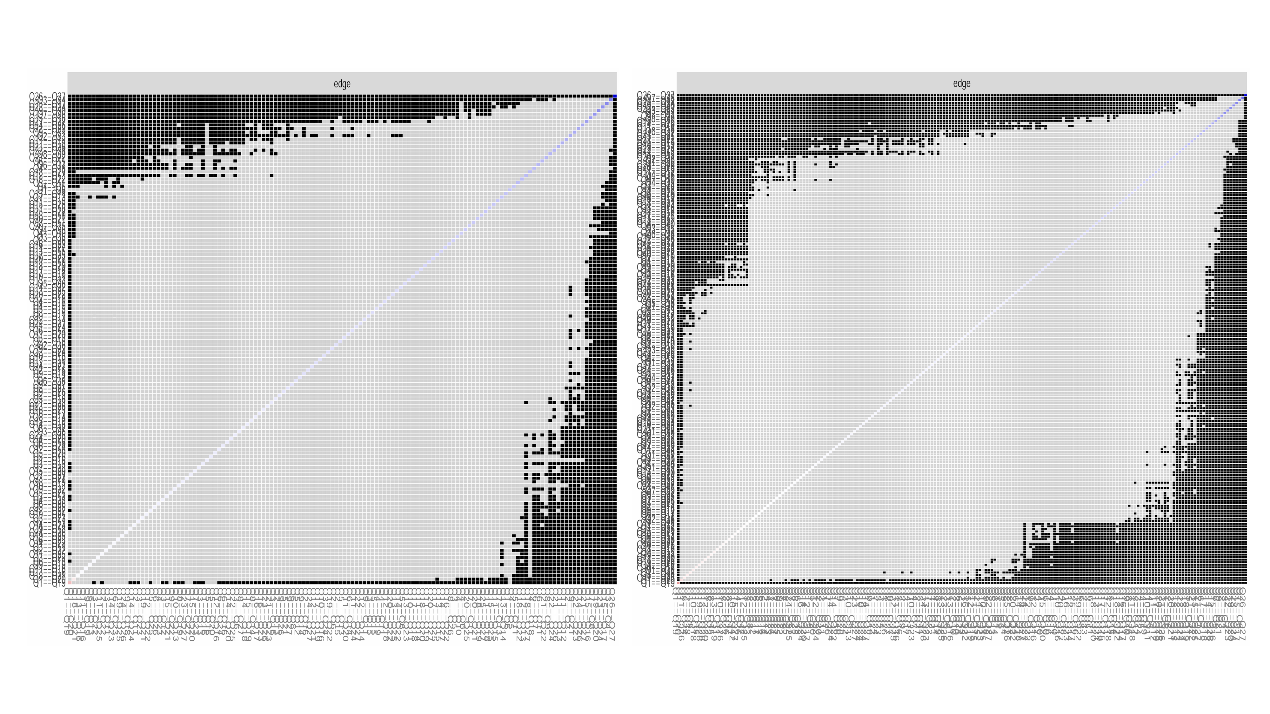


*Note:*This figure displays whether there is a statistically significant difference between the estimated weights of each pair of connections (edges) in the network, as assessed by a bootstrap test (1000 samples, 95% confidence intervals, p < 0.05). A black square indicates a significant difference between the weights of the corresponding pair of edges, while a grey square indicates no significant difference. Only pairs for which at least one edge had a non-zero weight are included. The diagonal is left blank as edges are not compared with themselves. The order of edges along the axes follows the same sequence as in the original network estimation. Both the horizontal and vertical axes represent all edges in the network.

*Figure S4* Testing the Disparity of Edges in the ASSQ-Ising Network(left) and the ASSQ-GGM Network(right)
